# Enteric Delivery of Probiotics: Challenges, Techniques, and Activity Assays

**DOI:** 10.3390/foods14132318

**Published:** 2025-06-30

**Authors:** Chunying Sun, Zhidong Zhang, Yantong Sun, Xueyuan Sun, Yan Jin, Jingwen Zhu, Jiaxin Yu, Tao Wu

**Affiliations:** State Key Laboratory of Food Nutrition and Safety, Food Biotechnology Engineering Research Center of Ministry of Education, College of Food Science and Engineering, Tianjin University of Science & Technology, Tianjin 300457, China; 18363263413@163.com (C.S.); z1250790195@163.com (Z.Z.); 18722407586@163.com (Y.S.); 15027606826@163.com (X.S.); 19838002496@163.com (J.Z.); 15602150842@163.com (J.Y.)

**Keywords:** probiotics, delivery systems, hydrogel, nanocoating, emulsion, core–shell microgel

## Abstract

Probiotics, as live microbial agents, play a pivotal role in modulating host microbiota balance, enhancing immunity, and improving gastrointestinal health. However, their application is hindered by critical challenges, such as inactivation during processing, storage, and gastrointestinal delivery, as well as low colonization efficiency. This article comprehensively reviews recent advances in probiotic delivery systems, focusing on innovative technologies, including hydrogels, nanocoatings, emulsions, and core–shell microgels. It provides an in-depth analysis of natural polyphenol-based nanocoatings and metal–phenolic network (MPN) single-cell encapsulation strategies for enhancing bacterial survival rates while highlighting the unique potential of microalgae-based bio-carriers in targeted delivery. Research demonstrates that well-designed edible delivery systems can effectively preserve probiotic viability and enable controlled intestinal release, offering novel strategies to reshape a healthy gut microbiome. While these systems show promise in maintaining probiotic activity and gut colonization, challenges remain in safety, scalable production, and clinical translation. Overcoming these barriers is crucial to fully harnessing probiotics for human health.

## 1. Introduction

Probiotics, defined as live microorganisms capable of regulating host biological functions and positively influencing health, have been widely employed to support human well-being and treat various diseases [1]. Recent studies demonstrate that probiotic supplementation can deliver multifaceted health benefits. These include maintaining intestinal homeostasis through microbiome modulation, enhancing immunological regulation, and synthesizing bioactive metabolites (e.g., short-chain fatty acids (SCFAs)) with demonstrated anti-inflammatory and cancer-preventive properties. Additionally, probiotics exhibit competitive exclusion effects against enteric pathogens [2,3,4]. A considerable number of functional probiotic-based preparations have demonstrated considerable potential in the treatment of a wide range of diseases that are difficult to treat. “Live microorganisms which when administered in adequate amounts confer a health benefit on the host”—this precise characterization, originally formulated by the World Trade Organization (WTO) and the Food and Agriculture Organization (FAO) in 2001, continues to serve as the benchmark definition [5]. Once the intestinal homeostasis of the human body is out of balance, it causes complications, such as inflammatory bowel disease (IBD), colon cancer, and other intestinal-related diseases, such as Alzheimer’s disease and depression [6]. The journey of probiotics—from traversing the gastrointestinal (GI) tract to colonizing the colon and engaging with microbial communities—creates unique biological challenges [7]. Consequently, developing effective strategies and methods to solve these problems is very challenging and necessary [8].

However, probiotic health benefits may not be fully realized as they are destroyed during food storage and digestion [9]. First, their intrinsic sensitivity presents challenges. Probiotics must withstand dual environmental stressors, including (1) variable physicochemical parameters during industrial processing and product storage, including thermal fluctuations, photonic exposure, hygrometric variations, and pH instability, and (2) aggressive gastrointestinal conditions post-consumption, particularly gastric acid corrosion (pH 1.5–3.5), enzymatic hydrolysis by proteases, and bile-acid-induced membrane destabilization. For example, *Lactobacillus acidophilus* exhibits moderate acid tolerance; however, a rapid decline in viability is observed during the later stages of fermentation, primarily due to the accumulation of organic acids and a significant drop in pH. *Bifidobacterium* is even less acid tolerant than *Lactobacillus*, ceasing growth below pH 5.0, whereas *Lactobacillus* stops growing below pH 4.0 [10]. Secondly, the therapeutic response is often inadequate, which can cause excessive cell death and suboptimal therapeutic outcomes. Moreover, practical application is hindered by bacterial virulence and invasiveness. These characteristics may give rise to severe adverse reactions. As a result, it is both highly challenging and essential to formulate effective strategies and approaches to address these issues [11].

Traditional probiotic encapsulation approaches like spray drying, freeze-drying, extrusion, and emulsification have demonstrated efficacy in boosting resistance to harsh external conditions. Nevertheless, each of these techniques harbors inherent limitations that impede their broader implementation [12]. Diverse embedding technologies have been devised to improve probiotics’ viability across processing, storage, transportation, and the entire gastrointestinal tract journey [13]. Probiotic embedding is a complete system that is influenced by a variety of factors, including embedding methods, materials, environmental conditions, and strain selection [14]. Sometimes, a combination of encapsulation materials gives better performance. Improved biocompatibility, biodegradability, easy processing procedures, and neutrality to probiotics are the main qualities to test while selecting materials to entrap probiotics [15]. To date, several methodologies have been developed for probiotic embedding. A comprehensive understanding of these embedding methods is imperative for the advancement of more sophisticated embedding techniques in the future [16].

**Figure 1:**
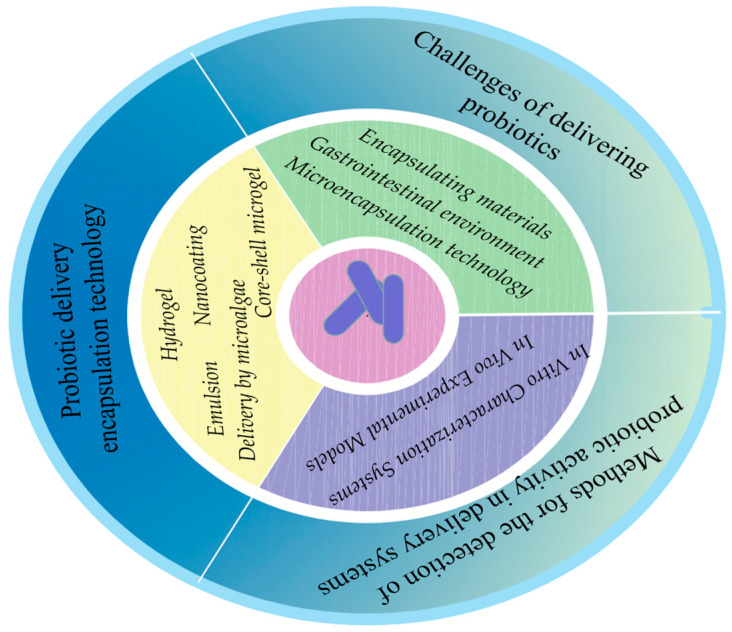
Enteric delivery of probiotics: challenges, techniques, and activity assays.

We systematically reviewed recent advances via a literature search (PubMed and Google Scholar, etc.). Using predefined criteria (study robustness, 2018–2025 timeframe), 133 articles were selected for in-depth analysis and synthesis. The purpose of this review is to enhance understanding of the topic. The article summarizes the current challenges of probiotic encapsulation, discusses the features and advantages of currently applied encapsulation methods, and delivers system probiotic activity assays (Figure 1). Finally, the article concludes by summarizing the potential prospects for the development of probiotic delivery systems.

## 2. Challenges of Delivering Probiotics

Despite advances in encapsulation technologies, maintaining probiotic viability during production remains a significant challenge. Current approaches struggle to simultaneously achieve high encapsulation efficiency, microbial survival rates, and industrial-grade quality standards, highlighting the urgent need for optimized methodologies [17]. In this section, we explore the challenges faced when developing probiotic delivery systems.

### 2.1. Encapsulating Materials

Firstly, the selection of encapsulating materials must be made based on their suitability for probiotic encapsulation. For instance, chitosan (CS), a natural, biocompatible, and biodegradable polymer, is derived from chitin through deacetylation. As the second most abundant natural polymer after cellulose, chitosan contains amino groups [18]. This characteristic not only simplifies its molecular and structural modification but also enhances its mechanical properties. However, it is important to note that chitosan has been observed to possess antimicrobial activity [19], and its direct use may result in the death of probiotics. Consequently, the development of probiotic delivery systems based on chitosan necessitates the identification of strategies to circumvent its inherent antimicrobial properties, thereby ensuring the preservation of its beneficial effects while maintaining the efficacy of the probiotic. Secondly, the wall materials for encapsulation must ensure targeted release in the colon while withstanding diverse adverse environments. Substances ingested by humans have limited residence time in the gastrointestinal tract (GIT). Thus, even if prepared probiotic agents can resist various harsh bodily conditions, within this limited timeframe, they may only release minimal encapsulated probiotics in the colon or fail to release them at all—rendering them ineffective and unable to exert the beneficial effects of probiotics. Therefore, based on colonic environmental conditions, during in vivo transportation, wall materials are typically selected as either pH-responsive materials that trigger the release of encapsulated probiotics in colonic pH environments or enzyme-specific hydrolyzable materials that undergo degradation exclusively by enzymes produced by colonic microbes, thereby achieving targeted colonic delivery [20]. Finally, it is imperative to consider the biodegradability of the encapsulated material [21] and the physiological and pathological effects on the human body [22] when selecting probiotic delivery materials, subsequent to the successful colonization and proliferation of encapsulated probiotics in the human gut [23].

Many colloidal delivery systems designed for small molecules (e.g., vitamins, nutraceuticals) are incompatible with probiotics. This is because the particle size of these systems is generally less than 1 µm, whereas the size of microbial cells is typically between 1 and 10 µm. It is widely acknowledged that the larger the particle size of the microcapsule, the more favorable it is for the encapsulation and protection of the probiotic. It’s important to note that this may negatively impact the organoleptic quality of the food [24,25]. Probiotics play their beneficial clinical role only when the number of living bacteria reaches more than 10^6^ CFU/g [26]. Additionally, for commercial products to deliver health benefits, they typically need to contain active probiotics at concentrations greater than 6 to 7 log_10_ CFU/g. This requirement calls for high loading capacity in the colloidal delivery system. However, large microcapsules (>2.0 mm) cannot pass the pyloric sphincter, preventing viable probiotic delivery to the colon. Instead, they tend to disintegrate and release probiotics in the stomach, where the harsh acidic environment (with a pH of approximately 1–3) makes the microorganisms vulnerable to degradation [27].

### 2.2. Gastrointestinal Environment

Probiotics encounter extreme stressors in proximal digestive regions, particularly the gastric lumen and duodenal–jejunal segments. Probiotics are generally adapted to the pH levels found in the colon, which typically range from approximately 6 to 7 [28]. However, gastric fluids are usually highly acidic (with a pH of approximately 1–3), which can negatively impact the survival of many probiotic strains [29]. In particular, the strongly acidic environment of gastric fluids lowers the cytoplasmic pH of probiotics. The high concentration of hydrogen ions (H^+^) and the decreased activity of glycolytic enzymes within probiotics disrupt the F_1_F_0_-ATPase proton pump, a critical mechanism for probiotic survival under acidic conditions [30]. Elevated ionic strength, pepsin activity, and mechanical agitation further compromise probiotic viability [31,32]. In the small intestine, bile acids and digestive enzymes (lipase, protease, amylase, and so on) have also been demonstrated to affect the viability of probiotics [33]. It has been discovered that high bile acid levels in the small intestine, particularly following a high-fat diet, may significantly reduce the efficacy of multiple probiotic treatments [34]. Upon their arrival in the colon, probiotics are compelled to engage in competitive interactions with the resident gut microbiota, establish adhesion to the intestinal mucus layer through specific molecular mechanisms, and undergo successful colonization and proliferation within the microbial niche of the gastrointestinal tract [35,36]. These harsh environmental conditions represent challenges that encapsulated materials must overcome when delivering probiotics to the human body.

### 2.3. Microencapsulation Technology

Microencapsulation effectively protects probiotics, enabling colon delivery. The four primary conventional probiotic microencapsulation methods comprise spray drying, freeze-drying, extrusion, and emulsification [37]. These techniques have been shown to enhance the tolerance of probiotics to adverse environmental factors, including food component interactions, highly acidic pH conditions, and the action of digestive enzymes. This is accomplished by creating a protective layer around the probiotics, which shields them from adverse conditions. However, these techniques have limitations. Conventional microencapsulation techniques may require high costs and energy consumption, especially for industrial-scale applications. For instance, spray drying, despite its capacity for large-scale manufacturing, is energy-consuming. Additionally, conventional techniques encounter difficulties in regulating the size and size distribution of microcapsules, which can impact the release characteristics and bioavailability of probiotics. The emulsification method is generally considered suitable for industrial production, and probiotics demonstrate a high survival rate when prepared using this method. However, emulsified microcapsules exhibit broad, uncontrolled size distribution (1–100 μm) and irregular shapes [1]. To address these limitations, further exploration of novel microencapsulation techniques and materials is imperative to enhance the efficiency of probiotic protection, reduce production costs, and achieve more precise particle size control.

## 3. Probiotic Delivery Encapsulation Technology

Advances in probiotic delivery systems demonstrate multiple encapsulation techniques, enhancing survival and bioavailability. Current research focuses on boosting probiotic activity in foods and the human gut, with hydrogel, nanocoating, and emulsion technologies receiving prominent attention. These utilize distinct protective mechanisms to improve harsh environment survival and gut adhesion/colonization. Such advancements not only enhance bioavailability but also enable innovative food and drug delivery systems, as detailed in Table 1.

### 3.1. Hydrogel

Hydrogels are regarded as optimal materials for the encapsulation of living cells due to their properties that mimic natural extracellular matrices, such as biosafety, biocompatibility, and biodegradability [56,57]. These materials primarily consist of polysaccharides and proteins, including starch, gelatin, whey proteins, alginate (Alg), xanthan gum, chitosan (CS), and soybean isolate proteins [12,58,59]. The formation of stable network structures is achieved through covalent cross-linking and non-covalent interactions, including electrostatic interactions and hydrogen bonding. The three-dimensional network of hydrogels provides a suitable growth environment for living cells and protects them from the external environment. Hydrogels also exhibit adjustable stiffness, stimulus-responsive degradation, surface adhesion, and self-repairing properties, enabling broad applications in cell culture, tissue engineering, and drug/cell delivery [60]. In the domain of probiotic delivery, hydrogels have garnered particular attention as a safe and effective strategy. However, the rapid degradation of protein-based hydrogels within the gastrointestinal tract has been shown to result in low transport efficiency and bioavailability [61,62]. Conversely, polysaccharide hydrogels have the capacity to be degraded by the microbiota in the colon, thereby facilitating efficient delivery of probiotics [63]. Nevertheless, polysaccharide hydrogels often suffer from weak gelation properties, poor mechanical properties, and insufficient tolerance to strong acidic environments, leading to premature carrier degradation [24]. Moreover, the porous structure and nanocoating of hydrogels have proven ineffective in preventing the diffusion of hydrogen ions and bile salts. Therefore, it is essential to develop hydrogel carriers with adequate mechanical strength, effective pH responsiveness, and improved mucosal adhesion to protect probiotics.

**Figure 2:**
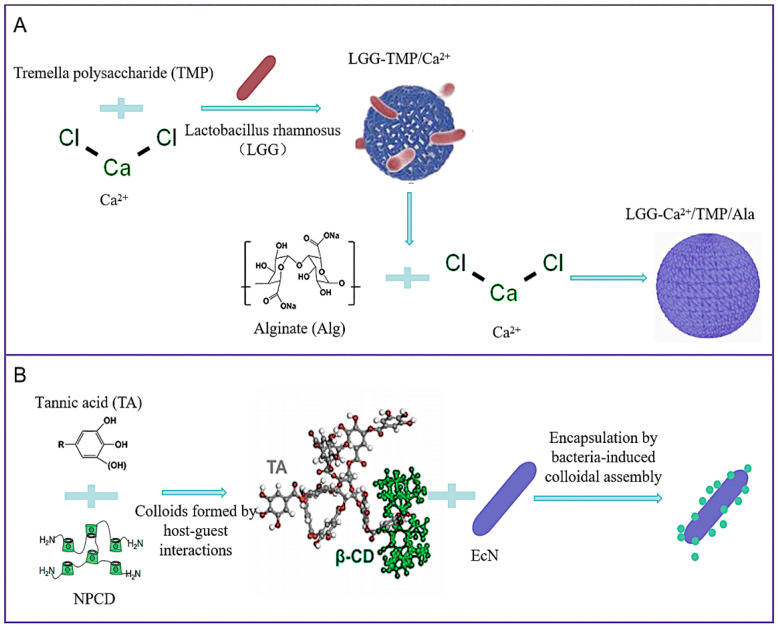
(**A**) Demonstration of a synthetic procedure for LGG-Ca^2+^/TMP/Alg, alginate (Alg) and Tremella polysaccharide (TMP), induced by calcium ions. LGG: *Lactobacillus rhamnosus*. (**B**) Probiotic encapsulation by bacteria-induced colloidal assembly; mixing of ethylenediamine (EDA)-modified poly-β-cyclodextrin (PCD) with tannic acid (TA) to form NPCD-TA colloids (NTc). EcN: *Escherichia coli Nissle 1917*.

Alginate (Alg), a natural anionic polysaccharide, is frequently employed as a wall material for probiotics due to its anti-gastric acidity, non-toxicity, good biocompatibility, and low cost [64]. Alginate (Alg) is capable of being degraded by intestinal microorganisms, exhibiting non-immunogenicity, mucosal adhesion, and other excellent biomedical properties [65]. In practical applications, the high porosity of a single alginate gel network is difficult to withstand harsh environments due to its unstable structure. Therefore, when constructing alginate carriers, it is usually necessary to introduce other gels for cross-linking to improve their performance. Tremella polysaccharide (TMP) is an acidic heteropolysaccharide extracted from the Tremella fruiting body. It exhibits excellent water retention properties, thereby providing a relatively suitable osmotic pressure environment for probiotics. Its pH responsiveness facilitates electrostatic interactions between Tremella polysaccharide (TMP) and alginate (Alg) molecules, resulting in the formation of stable gels. Lulu Chu et al. [38] induced Alg-TMP to form a gel shell using external calcium ions. In that study, a bilayer hydrogel protective carrier, Alg-Ca^2+^-TMP shell, was designed, which effectively resisted the erosion of strong acids and bile salts and protected the probiotics (Figure 2A). The carrier exhibited excellent gel properties, thermal stability, and pH responsiveness.

Pectin, an anionic polysaccharide, is frequently utilized as an encapsulation material due to its low cost, wide distribution worldwide, high biocompatibility, and good adhesion properties [66]. However, hydrogel beads prepared from sodium alginate/pectin are porous, leading to the rapid diffusion of the matrix and reducing the barrier to unfavorable conditions. To solve this problem, Yunsi Guo et al. [39] enhanced the thermal stability and gastrointestinal stability of microcapsules through high-efficiency vibration technology (HEVT) and the addition of antacids, such as calcium carbonate (CaCO_3_) nanocrystals. High-efficiency vibration technology (HEVT) generates homogeneous microcapsules by vibrating a laminar jet at a specific frequency, causing it to rupture [67]. Unlike traditional methods like high-shearing homogenization, which can damage probiotics through excessive shear force, high-efficiency vibration technology (HEVT) operates under minimal shear stress. This significantly reduces cell damage during microcapsule formation. While innovative for anti-acid microcapsule design, the study’s single-model approach and simplified conditions limit conclusion generalizability and application value.

In natural environments, extracellular matrices (ECM) surrounding bacteria provide resistance to harsh conditions, including extremes of pH and ultraviolet light (UV), as well as adhesive properties that promote bacterial colonization and adaptive capabilities that support sustained bacterial proliferation [68]. Chong Zhang et al. [40] proposed a bacterial-induced encapsulation strategy involving the mixing of ethylenediamine (EDA)-modified poly-β-cyclodextrin (PCD) with tannic acid (TA) to form NPCD-TA colloids (NTc). These colloids then assemble into artificial extracellular matrices (ECM) surrounding the bacteria, thereby mimicking the structural and functional features of bacterial extracellular matrices (ECM) (Figure 2B). This strategy is expected to be replicated in a variety of microorganisms [69]. The NPCD-TA colloid (NTc) relies on non-covalent host–guest interactions and may dissociate during long-term storage or in complex physiological environments (e.g., by intestinal enzymes, pH fluctuations) [70]. The study did not assess the shelf-life stability of the encapsulated probiotic formulation.

In recent years, studies have demonstrated that the delivery of the specific probiotics to the intestine using pH-sensitive materials to encapsulate could improve the survival of probiotics in the gastrointestinal (GI) tract [71]. Based on the fact that the bacterium cannot pass through the pore from the encapsulating matrix due to the larger size, the probiotic’s release was further depressed, obviously due to the compact and dense structure of the carrier in these studies, although survival showed vast improvement. Therefore, enhancing the disintegration intensity is the most frequent solution to improve the release profile. A growing body of research demonstrates that many materials can be specifically degraded by enzymes in the intestine while remaining intact in the stomach. It has been confirmed that protamine can be enzymatically hydrolyzed by trypsin, and calcium ions will be utilized in the process of enzyme digestion and lead to the disintegration of the calcium alginate matrix [72]. Chitosan (CS) can be specifically hydrolyzed by enzymes produced by colonic microorganisms [73]. This peculiar property has great potential for probiotics’ oral delivery. Based on such properties, Qikun Cheng et al. [41] designed and constructed an enzyme-triggered fuse-like microcapsule. By design, the microcapsule can protect probiotics from acid and bile and disintegrate layer by layer in response to trypsin. This formulation has excellent performance in the whole process of viability, release, and adhesion of probiotics and huge potential for colon-targeted oral delivery for probiotics. However, inter-individual variation in trypsin activity is large, and in groups with insufficient trypsin secretion, microcapsules may not disintegrate in a timely manner, resulting in delayed or insufficient release of probiotics.

Hydrogels are one of the core oral delivery carriers for probiotics due to their biocompatibility, mild processing, and precise intestinal targeting [74,75]. However, structural instability may trigger mechanical collapse, while inefficient barrier protection causes premature probiotic leakage in gastric acid. Future research must integrate multidimensional innovations to overcome these limitations.

### 3.2. Nanocoating

The field of coating technology has a significant role in enhancing the activity of probiotics in food products and the human gut. This nanocoating technology does not embed multiple probiotic cells in a certain matrix but rather coats a layer of material on the surface of a single probiotic cell, which can significantly enhance the bioavailability of probiotics. The nanocoating forms a protective layer through cross-linking driven by interactions like hydrogen bonding, electrostatic forces, van der Waals forces, and Schiff base reactions. This multi-interaction approach leverages the synergistic effect of diverse forces to establish a stable, adaptive, and multifunctional network structure. This protective layer can effectively shield probiotics from physical and biological damage and maintain their biological functions in harsh environments [76]. In addition, these nanocoatings can enhance the adhesion of probiotics to the intestinal mucosal epithelial tissue, preventing them from easily detaching from the intestinal mucosal surface [77].

**Figure 3:**
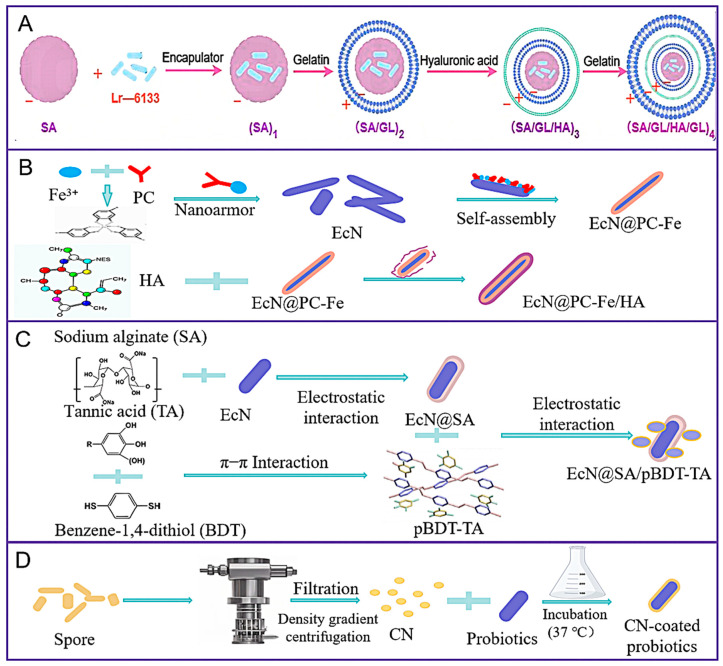
(**A**) Demonstration of a synthetic procedure for (SA/GL/HA/GL)_4_; (SA/GL)_2_, (SA/GL/HA)_3_, and (SA/GL/HA/GL)_4_ multilayered microcapsules were sequentially synthesized by alternating the deposition of gelatin (GL) and hyaluronic acid (HA) solutions on the core of sodium alginate (SA) microcapsules. Lr-6133: *Lactobacillus. rhamnosus 6133*. (**B**) Demonstration of a synthetic procedure for EcN@PC-Fe/HA; the first layer was a metal–phenolic network (MPN) formed by proanthocyanidins (PC) and Fe^3+^ (EcN@PC-Fe), and the second layer was high-molecular-weight hyaluronic acid (HMW-HA) (EcN@PC-Fe/HA). EcN: *Escherichia coli Nissle 1917*. (**C**) Demonstration of a synthetic procedure for EcN@SA/pBDT-TA; the pBDT-TA were prepared through self-polymerization of benzene-1,4-dithiol (BDT) with tannic acid (TA), followed by layer-by-layer coating with sodium alginate (SA) on the surface of EcN and the final construction of a synergistic platform of EcN@SA-pBDT-TA. EcN: *Escherichia coli Nissle 1917*. (**D**) Demonstration of a synthetic procedure for CN-coated probiotics; spores were squeezed back and forth to form CN, and, finally, CN adsorbs to the surface of probiotics.

The layer-by-layer (LBL) self-assembly method is a classic nanocoating embedding approach that encapsulates living biological cells or other microorganisms by successively adsorbing functional components with opposite charges, thereby preparing the cells for long-term storage. Layer-by-layer (LBL) technology, with the advantages of lower cost and the ability to produce multilayers, has a wide range of application potential in the fields of medicine, food, cosmetics, textiles, and agriculture [78]. The formation of the multilayer structure of layer-by-layer (LBL) technology is driven by various intermolecular interactions, such as hydrogen bonds, electrostatic interactions, covalent interactions, and hydrophobic interactions [79]. Among them, electrostatic interactions are the most common driving force for forming the multilayer structure embedded with probiotics [80]. For example, Leran Wang et al. [42] encapsulated probiotics with gelatin (GL) and hyaluronic acid (HA) through the layer-by-layer (LBL) assembly technique (Figure 3A) to enhance the activity and adhesion of probiotics in the intestinal mucosa.

In a recent development, Pan et al. [43] reported a single-cell nanocoating technology based on a metal–phenolic network (MPN) consisting of tannic acid (TA) and FeIII. The metal–phenolic network (MPN) is called ‘nanoarmor’. This nanocoating has been shown to protect probiotics from various types of antibiotics, particularly when probiotics are administered in enteric capsules following oral ingestion. High-molecular-weight hyaluronan (HMW-HA) is a negatively charged glycosaminoglycan biopolymer with anti-inflammatory properties and the ability to specifically bind to the Cluster of Differentiation 44 (CD44) receptor. Utilizing these properties, Limeng Zhu et al. [44] developed a layer-by-layer nanocoating strategy (Figure 3B) for targeted delivery of probiotics at inflammatory sites. This strategy employs a first layer of the metal–phenolic network (MPN) consisting of procyanidine (PC, composed of flavan-3-ol (epi)-catechin subunits, is a major dietary polyphenol found in abundance in fruits, vegetables, nuts, legume seeds, and cereal grains with a wide range of health benefits) and FeIII ions (EcN@PC-Fe), as well as a second layer incorporated into the first layer of HMW-HA (EcN@PC-Fe/HA) under cell-compatible conditions. Introducing the high-molecular-weight hyaluronan (HMW-HA) framework onto this responsive and degradable metal–phenolic network (MPN) coating is expected to enable probiotics to achieve not only extremely strong resistance to harsh environmental conditions but also site-specific targeted delivery and regulation capabilities.

However, nanocoatings prepared through layer-by-layer (LBL) assembly, while protecting probiotics and enhancing intestinal mucosal adhesion, may also impair the organoleptic quality of food products [81]. Furthermore, the traditional layer-by-layer (LBL) self-assembly single-cell encapsulation method requires cumbersome multilayer coatings, which not only increases the experimental complexity but also reduces the production efficiency [82].

The utilization of natural polyphenols in the formulation of nanocoatings represents a contemporary approach to encapsulating probiotics. Polyphenols are naturally derived bioactives that play a wide range of roles in regulating oxidative stress and inflammatory pathways and can be used as reactive oxygen scavengers [83,84,85]. Due to their unique structural characteristics, polyphenols, as an important type of bioactive substances, have attracted extensive attention [86]. However, to enhance therapeutic efficacy and minimize systemic exposure, their precise delivery to the lesion site is required. Recent years have seen significant advancements in nanotechnology, resulting in the creation of precise advanced delivery systems. Polyphenols have been used as building blocks for particle assembly and achieved better bioavailability and targeted delivery capabilities [87]. Tannic acid (TA) is a particularly promising candidate for nanoparticle engineering due to the presence of multiple hydroxyl groups and a phenolic structure. Qinglian Hu et al. [45] developed a novel ‘nanoarmour’ wrapping technique (Figure 3C), preparing nanostructured pBDT-TA via self-polymerization of aromatic dimercaptans (benzene-1,4-dithiol, BDT) and tannic acid (TA). The prepared pBDT-TA nanostructures and sodium alginate (SA) were coated layer by layer on the surface of *Escherichia coli Nissle 1917* (EcN) to construct the synergistic platform EcN@SA-pBDT-TA. This natural polyphenol-based supramolecular network enabled unicellular encapsulation of probiotics, providing excellent protection against oxidative and inflammatory stresses and achieving enhanced protection, colonic accumulation, and retention in an IBD mouse model. However, probiotics are required to undergo sodium alginate (SA) electrostatic adsorption (pH-sensitive) and secondary encapsulation of pBDT-TA nanoparticles for assembly. This process relies on electrostatic interactions and is susceptible to interference by intestinal pH fluctuations and ionic strength changes, which may lead to premature disintegration of the coating in the gastrointestinal tract (GIT).

In exploring the challenges of effective delivery and rapid colonization in probiotic therapy, researchers have proposed the use of spores, the dormant life form of probiotics, as a solution. Spores are considered to be particularly well-suited for probiotic delivery due to their robust resistance to stomach acid and their capacity to germinate within the gastrointestinal tract (GIT). Qingling Song et al. [46] developed a pioneering strategy to convert spore coatings into multifunctional coat nanomaterial (CN) through in vitro mechanical force extrusion (Figure 3D). The preparation method of coat nanomaterial (CN) coated probiotics can be widely applied to various probiotics, such as *Bacillus subtilis* (BS) and *Bacillus licheniformis* (BL). This method can maintain the integrity of the spore-coated components and shows significant advantages in the following aspects: tolerance to extreme environments, anti-inflammatory effects, epithelial barrier repair, and natural affinity for probiotics. However, this strategy is not without its limitations, including the uncontrollable efficiency of spore germination in vivo and the potential disruption of intestinal ion homeostasis by ions that may be secreted during spore germination. Consequently, the development of straightforward yet efficacious methodologies to enhance the overall fate of probiotics has emerged as a pivotal area of research.

As a frontier in probiotic delivery, nanocoating technology uses single-cell precision encapsulation to significantly enhance probiotic survival, intestinal colonization, and targeted therapeutic function in harsh environments—particularly for mucosal repair in conditions like inflammatory bowel disease (IBD) [82,88]. Although recognized as a key emerging pathway for high-precision delivery, clinical translation remains limited by three bottlenecks: process complexity, biosafety concerns, and scale-up barriers. Overcoming these barriers could enable nanocoating technology to advance from the lab to the clinic as a significant vehicle for precision microecological therapy.

### 3.3. Emulsion

Lipids are a fundamental component in the construction of food-grade delivery systems and extensively employed for the encapsulation of probiotics, owing to their biocompatibility, biodegradability, and nutritional properties. In addition, lipids can develop probiotic delivery systems by forming emulsions. An emulsion is a metastable system of two immiscible phases, with one dispersed as droplets in the other. When the dispersed phase is aqueous, the emulsion is called a water-in-oil (W/O) emulsion; conversely, it is an oil-in-water (O/W) emulsion [89]. If another phase is added again, a double emulsion can be obtained, such as a water-in-oil-in-water (W/O/W) or an oil-in-water-in oil (O/W/O) emulsion system [90]. Water-in-oil-in-water (W/O/W) emulsions are frequently employed to encapsulate ingredients with a high degree of hydrophilic properties [91], including polyphenols [92], minerals [93], peptides, and probiotics [48]. Probiotics are usually encapsulated in the internal aqueous phase of water-in-oil-in-water (W/O/W)-type emulsions, thereby protecting them from the influence of the external environment [94]. However, the stability of emulsions is a limiting factor in their practical application. The structure and nature of the interfacial layer in emulsions are critical to their stability and ability to protect probiotics. The application of imine chemistry has been shown to improve the properties of emulsion interfaces, thus enhancing their functional performance [95]. Gege Sun et al. [47] enhanced the stability of water-in-oil-in-water (W/O/W) emulsions by cross-linking proteins through aldehyde–amine reactions. This cross-linking reaction might occur at the oil–water interface or in the inner and outer aqueous phases. These emulsions were prepared with polyglycerol polyricinoleate (PGPR, a hydrophobic emulsifier) and isolated whey protein (WPI, a protein extracted from milk with high nutritional value that is easy to digest and absorb, containing a variety of active ingredients, etc.) as stabilizers, and the emulsions themselves have hydrophobic and hydrophilic properties. The incorporation of aldehyde lipids and whey protein (WPI) into the emulsion resulted in the successful preparation of an emulsion-based probiotic delivery system. It was demonstrated that cross-linking between cinnamaldehyde (CA, an aldehyde organic compound found in large quantities in plants, such as cinnamon) and whey protein (WPI) occurred in the presence of an optimized aldehyde mixture, resulting in the formation of a dense interfacial layer with unique interfacial properties in the presence of interfacial imine bonds [96]. This enables the water-in-oil-in-water (W/O/W)-type emulsion to protect probiotics from the simulated environment of the stomach and the small intestine. This research provides a new strategy for the protection and delivery of probiotics in food. However, it is worth noting that aldehydes may leave residual toxicity or off-flavors, limiting food applications.

Solid oil has been found to act as an effective heat absorber, and its phase change properties have been demonstrated to reduce thermal damage to probiotics during the process of spray drying. Furthermore, the addition of carbohydrates to solid oils has been shown to enhance the survival rate of probiotics and improve their glass transition temperature, thereby enhancing their activity during the processes of spray drying and transportation and within the digestive tract [97]. Prebiotics, a category of carbohydrates that are indigestible to humans, have been observed to stimulate the proliferation of probiotics. An increasing number of studies are exploring the potential of prebiotics as thermoprotectants, aiming to enhance the stability and activity of probiotics. Fioramonti et al. [98] demonstrated that double emulsions (W/O/W emulsions) exhibited a retention capacity of up to 84% of linseed oil following spray drying while maintaining stability during storage at −4 °C for a period of six months. This finding indicates that double emulsions have the potential to serve as effective carriers for probiotics, thereby mitigating the impact of heat stress on probiotics by utilizing the heat absorption capabilities of phase change materials in solid fats in conjunction with prebiotics. But, there are still drawbacks, as fat-fixing and heat absorption reduce heat damage, but the high-fat environment may prevent probiotic rehydration recovery.

Using *Lactobacillus rhamnosus GG* (LGG) as a model probiotic, Ming Yin et al. [48] studied the effect of embedding probiotics in a double emulsion to enhance their activity during spray drying and systematically explored their protective mechanism. In the study, four commercial prebiotics—inulin, fructo-oligosaccharide (FOS, a generic term for a series of homologous oligosaccharides in plants composed of linear chains of fructose units and linked by β (2→1) bonds), xylo-oligosaccharide (XOS, chains of xylose molecules linked by β1–4 bonds that are produced enzymatically through hydrolysis of xylan from oats, birch wood, or corn cobs), and galacto-oligosaccharide (GOS, a functional oligosaccharide with natural properties; its molecular structure generally consists of one to seven galactose groups attached to a galactose or glucose molecule)—were added to the double emulsion as wall materials, and the optimal prebiotics most suitable for LGG spray drying were further identified through a combination of spray drying and in vitro reculturing experiments. The results indicated that the addition of prebiotics significantly increased survival rates and enhanced the integrity of subcellular structures, especially inulin. The water-in-oil-in-water (W/O/W) emulsion combined with inulin has the lowest water activity and the highest glass transition temperature, which is conducive to the storage stability of probiotic spray-dried microcapsules. The research provides a valuable reference for the selection of wall materials to enhance the activity of probiotics during spray drying and storage through the dual emulsion encapsulation technology.

Despite the development and application of several types of water-oil-water (W/O/W) double emulsions for intestinal-targeted delivery, the protective role of probiotics in the gastrointestinal barrier requires further investigation [99]. Yi Li et al. [49] used sodium caseinate (NaCas) and kappa-carrageenan (κCar) as the external emulsifier and constructed a water-in-oil-in-water (W/O/W) bilayer emulsion system through the Maillard reaction. This emulsion was then compounded with a sodium alginate (SA) carboxymethyl chitosan (CMCS, which has excellent biological properties of chitosan and good water solubility) hydrogel shell to encapsulate *Lactobacillus rachrosus 76* (LR76) [100], and this system was named SCCS. The research not only offers a promising approach for the encapsulation of probiotics and their precise delivery to the intestines but also highlights the potential therapeutic value of probiotics in treating intestinal diseases. But, the SCCS systems are complex and have high costs of food-grade raw materials (e.g., κCar, CMCS). These can constrain its industrial production.

Lipid emulsions, as a representative of traditional liquid carriers in the field of probiotic delivery, have significant potential for oral delivery applications due to their good biocompatibility and amphiphilic co-loading ability (e.g., probiotic–prebiotic synergy) [29,101,102]. However, current studies are mostly limited to idealized laboratory environments and are not sufficiently adapted to real-world scenario, including emulsion destabilization due to dynamic fluctuations in gastric acid, phase separation due to changes in storage temperatures, and challenges to the consistency of encapsulation due to differences in strain diversity.

### 3.4. Core–Shell Microgel

**Figure 4:**
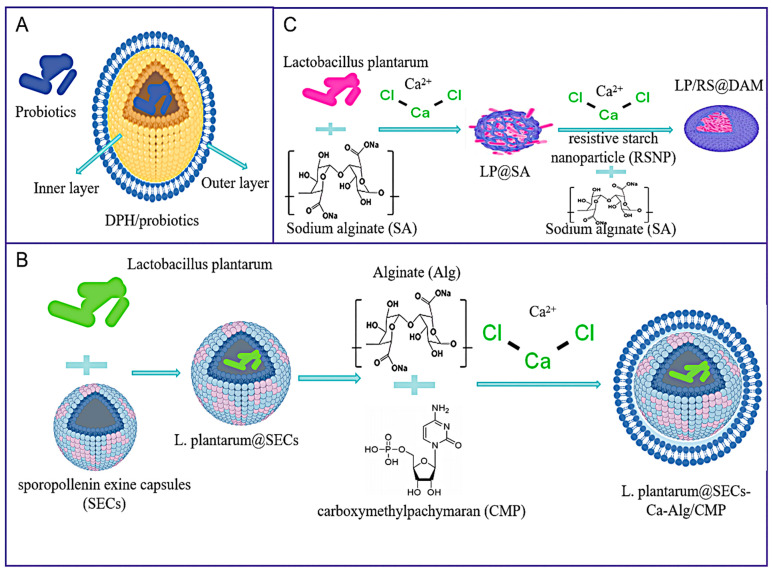
(**A**) Design rationale of the double-layer polysaccharide hydrogel (DPH). (**B**) Demonstration of a synthetic procedure for *L. plantarum*@SECs-Ca-Alg/CMP; the *L. plantarum*-encapsulating sporopollenin exine capsules (SECs) were evenly dispersed into a solution composed of carboxymethylpachymaran (CMP) and alginate (Alg). Then, the mixture was dropped into the CaCl_2_ solution. *L. plantarum*: *Lactobacillus plantarum*. (**C**) Demonstration of a synthetic procedure for LP/RS@DAM; the inner layer of sodium alginate (SA) was co-extruded with the outer layer of resistive starch nanoparticle (RSNP) and sodium alginate (SA) solution through centrifugal force into CaCl_2_ solution, cured, and collected to obtain LP/RS@DAM microspheres. LP: *Lactiplantibacillus plantarum*.

Core–shell microgel is typically composed of a core material and a shell material. The core–shell microgel provides a protective environment for probiotics by encasing them in a core material, such as sodium alginate (SA) or polysaccharides, and then forming a shell with substances, such as chitosan [103]. This structure confers protection for probiotics against deleterious environments, including elevated temperatures, humidity, and oxygen levels, as well as the action of gastric acid. Additionally, the strong interaction between the wall macromolecules of the core–shell microgel and intestinal mucins can enhance the adhesion and colonization of probiotics in the intestine. A further key role of core–shell microgels is to achieve the active release of probiotics in the gastrointestinal tract. For instance, Ca-alginate (Alg)/carboxymethylpachymaran (CMP, polysaccharides extracted from Poria cocos and further processed to obtain carboxymethylpachymaran, which possesses good immunomodulatory activity) gel as the shell has been shown to release probiotics following digestion within the gastrointestinal tract. In the research of Huang Wencan et al. [50], a double-layer polysaccharide hydrogel (DPH) was developed for the intestinal-targeted oral delivery of probiotics. The DPH comprised a bilayer network structure; carboxymethyl cellulose (CMCL, a carboxymethylated derivative of cellulose, which is the most important ionic cellulose glue) forms a hydrogen-bonded inner layer, while carboxymethyl chitosan (CMCS) covalently cross-links with dialdehyde alginate (DAA) to form an outer layer (Figure 4A). Probiotics encapsulated in the carboxymethyl chitosan (CMCL) layer showed significantly improved viability under extreme gastrointestinal conditions, with virtually unchanged activity. The DPH also exhibited excellent mucosal adhesion and enhanced intestinal colonization. Consequently, it is hypothesized that double-layer polysaccharide hydrogel (DPH) has the potential to serve as an effective alternative to conventional carriers for intestinal targeted delivery. Nevertheless, the double-layer polysaccharide hydrogel (DPH) requires precise control of the reaction conditions to achieve accurate release.

Sporopollenin exine capsules (SECs) derived from natural pollen are regarded as natural microcapsules for protecting sensitive biomolecules due to their multiple advantages, including larger inner cavities, tolerance to changes in temperature, pH, and ionic strength, and mucosal adhesion [104,105,106]. However, the presence of numerous pores on the sporopollenin exine capsule (SEC) surface is a double-edged sword, as it facilitates the encapsulation of probiotic bacteria while concurrently providing a conduit for digestive fluids in the gastrointestinal tract (GIT), thereby diminishing the activity of the probiotic bacteria. Ziyu Deng et al. [51] have proposed a novel core–shell structure for safeguarding and delivering probiotics in the food industry to address this challenge (Figure 4B). The core of this structure consists of sporopollenin exine capsules (SECs), with a Ca-Alg/CMP gel shell protecting the probiotics. This design has two main objectives: first, to boost the stability of probiotics during storage and lyophilization; second, to enable their controlled release in the gastrointestinal tract (GIT) for delivery to the human colon after commercial products undergo processing and storage. In the Ca-Alg/CMP shell, carboxymethylpachymaran (CMP) regulates shell swelling, microstructure, and probiotic release by modulating CMP-Alg hydrogen bonding. Carboxymethylpachymaran (CMP) also improves the shell layer’s thermal stability, which is crucial for industrial application. However, sporopollenin exine capsules’ (SECs) pore homogeneity is dependent on natural sources, with large batch variations that do not meet the standardization required for clinical translation.

In addressing the vulnerability of *Lactobacillus plantarum* (LP) to the highly complex ecosystem of intestinal flora, Mengyao Liu et al. [52] sought to deliver sufficient quantities of active LP to the colon (Figure 4C). However, challenges persist. They proposed a centrifugal-driven micro-nozzle system for the preparation of a double-layered core–shell alginate microcapsule (DAM), which can be used as a resistive starch nanoparticle (RSNP, a novel type of functional dietary fiber and a prebiotic that exhibits potential for the development of health-promoting foods), and LP as an effective carrier for dual delivery for the treatment of colitis. The system has demonstrated the ability to precisely load LP and resistive starch nanoparticles (RSNP) in the core and shell regions of the double-layered core–shell alginate microcapsules (DAM), respectively. However, there are still some problems, as the process of producing double-layer core–shell alginate microcapsules (DAM) prepared using a centrifugal-driven micro-nozzle system is complicated, the micro-nozzles are easily clogged, and the mechanical strength varies greatly between batches.

As a significant innovative solution in the field of probiotic delivery, the core–shell structure, through the synergistic optimization of materials and processes, provides probiotics with physical barriers and controlled release precision beyond the traditional encapsulation technology, which significantly improves their survival and bioavailability in gastrointestinal environments and opens up safer and more efficient delivery pathways [107,108]. However, the technology still faces key bottlenecks, including material intrinsic defects, insufficient adaptation to pathological environments, and barriers to scaling up. In the future, there is a need to develop smart-responsive shell layers (e.g., pH/enzyme dual-sensitive materials) and establish criteria for validation of pathology models rather than relying on complex multilayer structures.

### 3.5. Delivery by Microalgae

Porous hydrogels and nanoscale coatings cannot effectively block gastric hydrogen ions or intestinal bile salts. To advance probiotic delivery, we must overcome persistent encapsulation issues—low efficiency, toxic crosslinkers, and complex processing—by developing simpler, more efficient strategies.

Inspired by nature, the symbiotic relationship between bacteria and algae provides a novel idea to address these issues [109]. While symbiotic interactions form fundamental ecological frameworks with cross-domain applicability, their biomedical potential remains underexplored. Notably, microalgae-based systems have recently emerged as versatile bioplatforms demonstrating unique capabilities in photonic diagnostics, targeted drug delivery, hypoxic tumor modulation, and accelerated tissue regeneration, showcasing their transformative potential in precision medicine [110,111]. Studies have found that *Spirulina platensis* (SP) has characteristics of anti-inflammation, anti-oxidation, and regulating the intestinal flora [112]. In addition, *Spirulina platensis* (SP) has been proven to have a positive impact on the activity of various probiotics, including *Lactobacillus bulgaricus*, *Lactobacillus acidophilus*, *Lactococcus lactis*, and *Streptococcus thermophilus*. *Spirulina platensis* (SP) emerges as a superior vector for biologically derived drug delivery due to its distinctive properties, including its excellent biocompatibility, economic feasibility, extensive surface reactivity, precise phototactic navigation, and inherent motility. This unique combination of characteristics positions *Spirulina platensis* (SP) as an exceptional platform for targeted therapeutic applications [113]. In addition, the helical structure and larger size of *Spirulina platensis* (SP) help it be intercepted by intestinal villi [114,115], thereby prolonging the retention time of the probiotics loaded in the intestine. In addition, *Spirulina platensis* (SP) microalgae can produce specific biological enzymes (including superoxide dismutase (SOD)), which can eliminate superoxide anion free radicals (O2•−) and may increase the survival rate of probiotics in the inflamed intestine [116]. Therefore, delicately designing *Spirulina platensis* (SP) microalgae as a universal platform offers an innovative approach for probiotic delivery [117].

**Figure 5:**
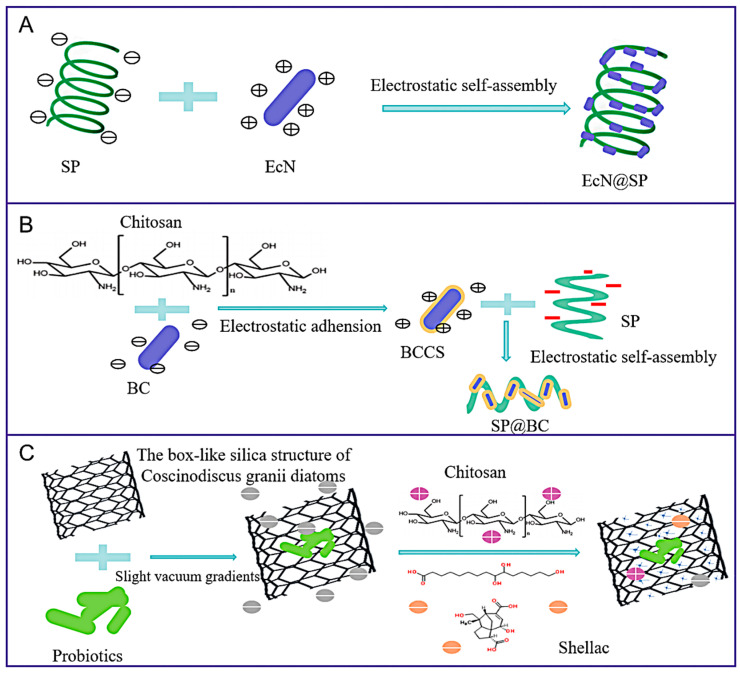
(**A**) Demonstration of a synthetic procedure for EcN@SP; by employing a straightforward one-step mixing method, EcN could be effectively incorporated onto *Spirulina platensis* (SP) to create a bacteria–microalgae symbiotic system (EcN@SP). EcN: *Escherichia coli Nissle 1917*. (**B**) Demonstration of a synthetic procedure for SP@BC; through the principle of electrostatic self-assembly, chitosan (Chitosan) is electrostatically adsorbed with the core substance *Spirulina platensis* (SP), and the composite structure SP@BC is finally formed. (**C**) Biosilica with pores and microscopic opening points for probiotics; electrostatic elements given by the surface silanol moieties of the biosilica, the ammonium moiety from chitosan (CS), and the carboxylate functions belonging to the acidic residues of Shellac (S).

Hui Huang et al. [53] proposed a natural delivery strategy that exploits the symbiotic relationship between bacteria and microalgae by employing *Spirulina platensis* (SP) as a delivery method. *Spirulina platensis* (SP) was used as a natural carrier for delivering *Escherichia coli Nissle 1917* (EcN), and the constructed bacteria–microalgae symbiotic system (EcN-SP) demonstrated higher EcN delivery efficiency in the treatment of inflammatory bowel disease (IBD) (Figure 5A). And, in order to further exploit the increased delivery efficiency of this symbiotic system, Zi-Yi Han et al. [54] developed a microalgae biomass-assisted probiotic delivery system (referred to as SP@BC) to enhance the efficacy of bacterial therapy in the treatment of inflammatory bowel disease (IBD) (Figure 5B). The SP@BC system was constructed through electrostatic adsorption of the chitose-coated probiotic strain EcN to the surface of *Spirulina platensis* (SP). After oral administration, due to the chitosan (CS) coating and the protective effect of *Spirulina platensis* (SP), this system demonstrated an enhanced bacterial survival rate. This biomimetic delivery system, designed by mimicking natural symbiosis mechanisms, demonstrates key strengths in production feasibility, biosafety, and therapeutic precision, showing particular promise for managing inflammatory bowel disease and gut dysbiosis syndromes.

Empty natural structures from microorganisms can act as bioreactors for producing nanomaterials. These examples include viruses [118] for metal nanoparticle synthesis, green microalgae [119] for the production of nanoparticles, and model bacteria [120] for the synthesis of gold nanomaterials. As an external membrane material, diatom frustules have been widely applied in fields like biomedicine [121], drug delivery [122], photonics [123], and imaging. Shellac (S) is an enteric polymer derived from the hardened secretion of the insect Kerria lacca found on trees in Asian countries [124], already exploited for its gastro-protective resistance. Danilo Vona et al. [55] presented an easy and sustainable way to use the box-like silica structure of Coscinodiscus granii diatoms as porous containers for living probiotics (Figure 5C). The process started with inducing bacteria to enter across the micropores present on the nanostructured surface of diatoms by means of slight vacuum gradients. After this loading, a combination of the natural soft Shellac (S) and chitosan (CS) polymers was used as embedding polymer matrices sealing the microalgae shells [125]. The combination of the abovementioned sealing polymers confers probiotics a certain resistance to harsh conditions, like simulated digestive solutions, storage, and thermal shocks, paving the way to an all-bio-based solution as bacterial supplements for future food.

As a bio-inspired innovation pathway in the field of probiotic delivery, microbial natural carriers have broken through the domain-limiting bottleneck of traditional artificial carriers through the dual protection mechanism of physical encapsulation and biochemical synergism, opening up a new pathway for natural–synthetic fusion for oral probiotic therapy [53,117]. However, the core contradiction lies in the fundamental conflict between the inherent uncontrollability of natural systems and the need for precise clinical delivery.

### 3.6. Summary

The latest advances in probiotic delivery encapsulation highlight five distinct strategies: hydrogels, nanocoatings, emulsions, core–shell microgels, and microalgae-based systems. As systematically summarized in Table 2, these technologies exhibit significant disparities in material composition and protective efficacy. However, they share common challenges in clinical translation, necessitating continued exploration for effective solutions.

## 4. Methods for the Detection of Probiotic Activity in Delivery Systems

The development of innovative probiotic delivery systems necessitates comprehensive evaluation across four critical parameters: structural integrity, physicochemical characteristics, functional efficacy, and microbial survival rates. Validation protocols typically employ integrated in vitro and in vivo approaches, where high-throughput screening using simulated gastrointestinal models provides preliminary data, while animal models enable translational validation of host–microbe interactions and targeted colonization efficiency. This tiered assessment strategy balances experimental throughput with biological relevance, addressing the trade-off between screening efficiency and physiological fidelity.

### 4.1. In Vitro Characterization Systems

The structural characterization of microcapsules is primarily conducted using analytical techniques, such as dynamic light scattering (DLS), scanning electron microscopy (SEM), transmission electron microscopy (TEM), and atomic force microscopy (AFM). These methods effectively show the particle size distribution, surface morphology, and nanoscale surface roughness of microcapsules. Fourier-transform infrared spectroscopy (FTIR) combined with nuclear magnetic resonance (NMR) spectroscopy enables precise elucidation of molecular interactions and chemical bonding mechanisms between components within microcapsules. For probiotic viability assessment, a Live/Dead BacLight Bacterial Viability Kit coupled with confocal laser scanning microscopy (CLSM) or flow cytometry allows for quantitative analysis of probiotic localization and survival rates in microgels—viable cells exhibit green fluorescence, while dead cells display red fluorescence [126]. Complementarily, methylene blue staining differentiates live cells via redox activity; viable cells reduce the dye to a colorless product, whereas dead cells retain blue staining [127].

Following ISO 2015 standards [128], the gastrointestinal tolerance of probiotic delivery systems is validated through in vitro digestion models. Specifically, the microcapsules need to be incubated in sequence in simulated saliva fluid (SSF, pH 6.8), simulated gastric fluid (SGF, containing pepsin, pH 1–2), and simulated intestinal fluid (SIF, containing bile salts and pancreatic enzymes, pH 5–7). Post-digestion viability is quantified via plate counting or flow cytometry [129,130].

### 4.2. In Vivo Experimental Models

Animal models serve as critical tools for evaluating in vivo probiotic delivery efficacy. Oral administration of fluorescently labeled engineered strains combined with in vivo imaging systems enables real-time tracking of probiotic distribution dynamics and colonization patterns in the murine gastrointestinal tract [131]. In disease models, dextran sulfate sodium (DSS)-induced colitis experiments demonstrated that microencapsulated probiotics significantly ameliorated colon length shortening (*p* < 0.01), reduced polyp count (*p* < 0.05), and normalized hematological parameters. Another study adopted a multi-model validation strategy; acid- and bile-tolerant *Lactobacillus* and *Bifidobacterium* strains (e.g., *L. plantarum T34* and *L. paracasei YJ10*) were first screened through simulated gastrointestinal fluids, followed by confirmation of their intestinal motility-enhancing effects in zebrafish constipation models and loperamide-induced murine constipation models, showing efficacy comparable to the standard *Lactobacillus rhamnosus GG* (LGG) strain [132].

Notably, significant interspecies differences exist between rodents and humans, as canine gastric emptying kinetics closely resemble human physiological characteristics, while porcine colonic anatomy shows high structural homology with humans. Although large animal models provide clinically relevant data, their implementation entails higher experimental costs and more complex ethical review processes.

## 5. Conclusions and Prospects

The emerging recognition of the gut microbiome’s pivotal role in host pathophysiology has consequently revolutionized therapeutic development towards precision-targeted oral vectors capable of shielding and transporting viable probiotics through harsh gastrointestinal barriers for colonic colonization. This review systematically analyzes the multifaceted challenges in engineering probiotic formulations, encompassing biological barriers and industrial manufacturing bottlenecks. This review subsequently examines pivotal encapsulation innovations designed to enhance probiotic viability under physiological stressors. To systematically evaluate delivery efficacy, standardized validation protocols incorporating in vitro simulated digestion models and in vivo colonization tracking methodologies are analyzed. Through optimized delivery architectures, these engineered systems potentiate probiotic functionality by modulating microbial ecosystems, thereby establishing host–microbe symbiosis critical for metabolic and immunological homeostasis.

Despite significant technological advancements, the clinical translation of probiotic delivery systems still faces multiple bottlenecks. (1) Probiotic delivery systems integrate materials via covalent or non-covalent bonds. Bond strength dictates functionality; excessive stability prevents timely probiotic release at target sites, while insufficient stability causes premature discharge before destination arrival. Both scenarios compromise precise delivery to diseased tissues. (2) Preparing probiotic delivery systems involves complex steps: probiotic cultivation, material preparation, carrier construction, and drug loading. Because laboratory research primarily relies on manual operations, achieving high automation and standardization is difficult, leading to significant variations in each preparation process. (3) The contradiction between drastic probiotic viability loss (>40% during lyophilization) in industrial-scale production and cost control necessitates developing economically viable stabilization solutions. (4) Clinical validation is insufficient, with only 12% of current systems demonstrating endoscopy-confirmed colonic delivery efficiency (<35% recovery rate), urgently requiring large-scale human trial data support. (5) Clinical applications require strict batch consistency, quality traceability, and stability testing; however, existing technology cannot meet these requirements, resulting in difficulties in translating findings from the laboratory to industrial production and clinical applications. (6) Co-encapsulation of small molecules (e.g., antibiotics) with probiotics is highly challenging because most probiotics are sensitive to antibiotics and cannot survive after co-administration.

To partially address these challenges, we suggest recommended future research directions for probiotic delivery systems, aiming to inspire researchers and developers to bridge critical technological gaps and resolve the aforementioned limitations. (1) Further develop novel encapsulation biomaterials (e.g., extracellular vesicles, fungal cell walls) and integrate advanced technological strategies—including microfluidics, biomineralization, and 3D/4D bioprinting—to achieve precision-controlled release and targeted delivery of probiotics in vivo through diversified pathways. (2) Integrate automated production technologies and intelligent equipment, such as microfluidic chip systems or high-precision bioreactors, to be deployed for large-scale probiotic cultivation. Real-time monitoring of critical cultivation parameters (temperature, pH, dissolved oxygen/DO) via integrated sensor networks minimizes human operational errors. (3) Through strategic integration of bio-based protective material innovation, stepwise phase-transition control, and cellular tolerance induction, this three-dimensional synergy achieves breakthrough enhancement of probiotic lyophilization viability within an economically viable framework. (4) By dynamically tracking probiotic colonic localization via non-invasive smart capsules and leveraging rapid organ-on-a-chip prescreening, this approach cost-effectively generates comprehensive clinical evidence to overcome current limitations in delivery efficiency validation. (5) Standard Operating Procedures (SOPs) are concurrently established, coupled with data-driven optimization of process parameters and online quality monitoring systems, ensuring batch-to-batch consistency in probiotic viability, payload efficiency of functional compounds, and performance of the delivery system. (6) While current genetic engineering strategies confer antibiotic resistance to probiotics to address coexistence challenges, their application remains constrained by biosafety concerns and regulatory constraints. There is a critical need to develop innovative encapsulation systems that enable synergistic co-delivery while maintaining physical segregation between probiotics and antibiotics, with the core challenge being the construction of spatially partitioned functional microenvironments.

Notwithstanding these translational barriers, engineered probiotics retain significant therapeutic potential for addressing complex disease pathologies. Successful resolution of these barriers would enable precision delivery platforms to offer novel therapeutic modalities, particularly in combating inflammatory bowel disease, antibiotic-associated dysbiosis, and metabolic disorders. Such advancements could fundamentally transform microbiome-targeted interventions, bridging critical gaps between microbial ecology research and clinical precision therapeutics.

## Figures and Tables

**Table 1:** Summary of each embedding method.

Types	Materials	Strains	References
Hydrogel	Alginate, tremella polysaccharide	*Lactobacillus rhamnosus*	[38]
	Alginate, pectin, CaCO_3_ nanocrystals	*Lactobacillus rhamnosus GG*	[39]
	Poly-β-cyclodextrin, tannic acid	*Escherichia coli Nissle 1917*	[40]
	Alginate, protamine	*Escherichia coli MG1655*	[41]
Nanocoating	Hyaluronan, gelatin	*Lactobacillus rhamnosus 6133*	[42]
	Tannic acid, FeIII	*Escherichia coli Nissle 1917*	[43]
	High-molecular-weight hyaluronan, procyanidine, FeIII	*Escherichia coli Nissel 1917*	[44]
	Tannic acid, benzene-1,4-dithiol, sodium alginate	*Escherichia coli Nissle 1917*	[45]
	Spore coat nanomaterial	*Bacillus coagulans*	[46]
Emulsion	Whey protein isolate, polyglyceryl polyricinoleate, cinnamaldehyde, citronellal, valeraldehyde, soybean oil	*Lactobacillus plantarum*	[47]
	Solid oil, inulin, fructo-oligosaccharide, Galacto-oligosaccharide, xylo-oligosaccharide	*Lactobacillus rhamnosus GG*	[48]
	Sodium caseinate, kappa-carrageenan, sodium alginate, carboxymethyl chitosan	*Lactobacillus rhamnosus 76*	[49]
Core–shell microgel	Carboxymethyl cellulose carboxymethyl chitosan, dialdehyde alginate	*Lactobacillus plantarum*	[50]
	Sporopollenin exine capsules, Ca-alginate, carboxymethylpachymaran	*Lactobacillus plantarum*	[51]
	N-Propanol, sodium alginate, resistant starch nanoparticles	*Lactiplantibacillus plantarum*	[52]
Delivery by microalgae	*Spirulina platensis*	*Escherichia coli Nissle 1917*	[53]
	Chitosan, *spirulina platensis*	*Escherichia coli Nissle 1917*	[54]
	Coscinodiscus granii diatom, shellac, chitosan	*Lactobacillus rhamnosus IMC 501^®^*, *Lactobacillus paracasei* *IMC 502^®^*	[55]

**Table 2:** Probiotic delivery: types, advantages and disadvantages, and current status of research.

Types	Advantages	Disadvantages	Status	References
Hydrogel	Excellent biocompatibility, mild preparation conditions, and ability to precisely regulate intestinal targeted release	Lack sufficient mechanical strength and stability	One of the core solutions for oral delivery systems	[74,75]
Nanocoating	Superior resistance to extreme conditions and stable adhesion	Process complexity and biosafety concerns	A frontier in the field of probiotic delivery	[82,88]
Emulsion	Good biocompatibility and amphiphilic co-loading ability Good physical isolation	Phase separation due to changes in storage temperatures	Traditional liquid carriers in the field of probiotic delivery	[29,101,102]
Core–shell microgel	Physical barriers and controlled release precision	Material intrinsic defects and insufficient adaptation to pathological environments	A significant innovative solution in the field of probiotic delivery	[107,108]
Delivery by microalgae	Physical encapsulation and biochemical synergism	The inherent uncontrollability of natural systems	A bio-inspired innovation pathway in the field of probiotic delivery	[53,117]

## Data Availability

No new data were created or analyzed in this study. Data sharing is not applicable to this article.

## Abbreviations

SCFAs: short-chain fatty acids; WTO: World Trade Organization; FAO: Food and Agriculture Organization; IBD: inflammatory bowel disease; GI: gastrointestinal; CS: chitosan; Alg: alginate; TMP: Tremella polysaccharide; HEVT: high-efficiency vibration technology; CaCO_3_: calcium carbonate; ECM: extracellular matrices; EDA: ethylenediamine; PCD: poly-β-cyclodextrin; TA: tannic acid; NTc: NPCD-TA colloids; LBL: layer-by-layer; GL: gelatin; HA: hyaluronic acid; MPN: metal–phenolic network; HMW-HA: high-molecular-weight hyaluronan; CD44: Cluster of Differentiation 44; PC: procyanidine; BDT: benzene-1,4-dithiol; pBDT-TA: self-polymerizing aromatic dimercaptans (BDT) and tannic acid (TA); SA: sodium alginate; EcN: Escherichia coli Nissle 1917; GIT: gastrointestinal tract; CN: coat nanomaterial; W/O: water-in-oil; O/W: oil-in-water; W/O/W: water-in-oil-in-water; O/W/O: oil-in-water-in oil; PGPR: polyglycerol polyricinoleate; WPI: whey protein; CA: cinnamaldehyde; LGG: Lactobacillus rhamnosus GG; FOS: fructo-oligosaccharide; XOS: xylo-oligosaccharide; GOS: galacto-oligosaccharide; NaCas: sodium caseinate; κCar: kappa-carrageenan; CMCS: carboxymethyl chitosan; LR76: Lactobacillus rachrosus 76; CMP: carboxymethylpachymaran; DPH: double-layer polysaccharide hydrogel; CMCL: carboxymethyl cellulose; DAA: dialdehyde alginate; SECs: sporopollenin exine capsules; CMP: carboxymethylpachymaran; LP: Lactobacillus plantarum; DAM: double-layered core–shell alginate microcapsules; RSNP: resistive starch nanoparticle; SP: Spirulina platensis; BC: Bacillus coagulans; SOD: superoxide dismutase; S: Shellac; DLS: dynamic light scattering; SEM: scanning electron microscopy; TEM: transmission electron microscopy; AFM: atomic force microscopy; FTIR: Fourier-transform infrared spectroscopy; NMR: nuclear magnetic resonance; CLSM: confocal laser scanning microscopy; SSF: simulated saliva fluid; SGF: simulated gastric fluid; SIF: simulated intestinal fluid; DSS: dextran sulfate sodium. SOPs: Standard Operating Procedures.
